# Dynamic reorganization of task-related network interactions in post-stroke aphasia recovery

**DOI:** 10.1093/brain/awaf036

**Published:** 2025-01-30

**Authors:** Zhizhao Jiang, Philipp Kuhnke, Anika Stockert, Max Wawrzyniak, Ajay Halai, Dorothee Saur, Gesa Hartwigsen

**Affiliations:** Max Planck Institute for Human Cognitive and Brain Sciences, 04103 Leipzig, Germany; Department of Neurology, Leipzig University Medical Center, 04103 Leipzig, Germany; Wilhelm Wundt Institute for Psychology, Leipzig University, 04109 Leipzig, Germany; Max Planck Institute for Human Cognitive and Brain Sciences, 04103 Leipzig, Germany; Wilhelm Wundt Institute for Psychology, Leipzig University, 04109 Leipzig, Germany; Department of Neurology, Leipzig University Medical Center, 04103 Leipzig, Germany; Department of Neurology, Leipzig University Medical Center, 04103 Leipzig, Germany; MRC Cognition and Brain Science Unit, University of Cambridge, Cambridge, CB2 7EF, UK; Department of Neurology, Leipzig University Medical Center, 04103 Leipzig, Germany; Max Planck Institute for Human Cognitive and Brain Sciences, 04103 Leipzig, Germany; Wilhelm Wundt Institute for Psychology, Leipzig University, 04109 Leipzig, Germany

**Keywords:** language, aphasia, language network, multiple-demand network, effective connectivity, dynamic causal modelling

## Abstract

Post-stroke aphasia is a network disorder characterized by language impairments and aberrant network activation. While patients with post-stroke aphasia recover over time, the dynamics of the underlying changes in the brain remain elusive. Neuroimaging work demonstrated that language recovery is a heterogeneous process, characterized by varying activation levels in several regions of the left-hemispheric language network and the domain-general bilateral multiple-demand network. Crucially, this activation seems to depend on the time elapsed since stroke and the lesion location. Yet, beyond task-related brain activation, the degree and nature of interactions between regions of the language and the multiple-demand network are not well understood. In this longitudinal functional neuroimaging study, we characterized task-related functional interactions between regions of the language and the multiple-demand network during language processing. We hypothesized that interactions between language regions and between language and multiple-demand regions should change over time and depend on lesion location.

We compared changes in effective connectivity in patients with left-hemispheric frontal or temporo-parietal stroke (*n* = 17 per group) and healthy controls (*n* = 17) with Dynamic Causal Modelling. All patients repeatedly underwent an auditory sentence comprehension paradigm during functional neuroimaging in the acute (≤1 week), subacute (1–2 weeks) and chronic (>6 months) phases after stroke.

We found overall increased task-related connectivity from regions of the multiple-demand to the language network across patients, resembling the principal pattern of task-related interactions in controls. Early facilitation from multiple-demand to language regions correlated with later language improvement across both groups. Crucially, recruitment of specific connections from regions of the multiple-demand to language network depended on lesion location and changed over time. In the chronic phase, patients with frontal stroke showed facilitatory modulation from the right dorsolateral prefrontal cortex, while patients with temporo-parietal stroke integrated the supplementary motor area/dorsal anterior cingulate cortex. Besides this across-network reorganization, facilitatory connectivity between regions of the language network emerged in all patients in the subacute phase. Additionally, patients with frontal stroke showed facilitatory influences from the right lesion homologue to the remaining undamaged left inferior frontal cortex in the acute phase.

Collectively, we provide first evidence that functional interactions of regions within and across the language and the multiple-demand network facilitate aphasia recovery. The identified dynamic reorganization principles over the time course of recovery may inform the future use of personalized treatment protocols with neurostimulation in aphasia rehabilitation. These protocols should be tailored to the individual lesion location and recovery phase.

## Teaser

Jiang *et al.* investigated changes in network interactions during language recovery in patients with post-stroke aphasia. Their findings suggest that successful language recovery involves reorganization both across networks - from multiple-demand regions to language areas - and within the language network itself.

## Introduction

Language is organized in distributed networks in the human brain. Numerous neuroimaging studies show that different language functions mainly rely on task-related activations in left-hemispheric inferior frontal as well as anterior and posterior temporal brain regions.^1^ These regions strongly interact during language processing^2^ and constitute the left-lateralized language network (LN). Activation of the language network is associated with diverse linguistic operations, including the processing of the meaning and sound of words, as well as the structure of sentences.^3^ Aside from the recruitment of core LN areas, different language tasks often engage additional domain-general regions. These regions include bilateral parietal cortices, prefrontal and pre-supplementary motor areas and adjacent dorsal anterior cingulate cortices of the multiple-demand network (MDN). The MDN is linked to attention, executive control, response inhibition, and working memory.^4,5^ Activation of the MDN complements the LN, especially under challenging processing conditions.^6-9^ Importantly, functional interactions not only occur between regions ‘within’ the LN or MDN but also ‘across’ these networks.^10^ For example, the left dorsolateral prefrontal cortex and pre-supplementary motor area were shown to functionally interact with frontal and temporal language areas in various speech and language tasks.^11,12^

Stroke-induced brain lesions to the LN often cause aphasia, from which patients can recover over time.^13^ In recent years, numerous cross-sectional neuroimaging studies have measured the magnitude of (re-)activation of brain regions to understand the neural mechanisms underlying aphasia recovery. Collectively, these studies emphasize that language recovery after stroke engages both the LN and MDN.^14^ In the chronic phase after stroke, patients activate perilesional and undamaged left-hemispheric regions of the LN during different language tasks.^15-17^ Additional activations of the prefrontal right-hemispheric lesion homologue and bilateral MDN regions also contribute to language recovery.^18-21^ Specifically, the MDN seems to support language recovery when patients are more severely affected^22^ and suffer from larger lesions,^23^ which may aid the restitution of undamaged areas in the LN.^24,25^

Fewer studies have explored longitudinal activation changes during aphasia recovery.^25-29^ Overall, these studies support the hypothesis that both LN and MDN contribute to language recovery, with the involvement of specific areas and the timing of their activation depending on the phase after stroke and the lesion location.^25,27^ These findings indicate that language reorganization takes place in brain regions of pre-existing networks which are also engaged in healthy participants. Notably, task-based functional neuroimaging analyses that measure regional activations provide limited insights into the nature and degree of the interactions within and between networks and potential changes over time. Accordingly, aside from activity changes, it is reasonable to assume that increases in the functional interactions between regions of the LN and MDN also contribute to aphasia recovery.

Indeed, more recent neuroimaging studies consider post-stroke aphasia as a network disorder, emphasizing the impact of disturbed interactions between key regions in the LN^30^ and between the LN and the MDN.^31^ Using resting-state connectivity measures, Siegel et al.^31^ showed that post-stroke aphasia not only results from local dysfunction of left hemisphere language regions, but also from disturbance of bilaterally distributed networks.^31^ Although resting-state connectivity has provided insight into intrinsic network organization, the directionality of task-related connectivity changes from the intrinsic network can only be addressed by effective connectivity measures. Yet very few studies investigated task-related effective connectivity in post-stroke aphasia to date and all of them were performed in the chronic phase. These include studies on treatment-induced changes in effective connectivity among language and multiple-demand regions^32-34^ and spontaneous effective connectivity changes during semantic processing.^35-37^ Collectively, these studies show that network interactions during language processing in chronic stroke patients are characterized by increased connectivity from multiple-demand to language regions. However, it remains uncertain whether these interactions depend on lesion location and change over the course of recovery, thereby facilitating behavioural improvement. Demonstrating this in patients with post-stroke aphasia would substantiate the hypothesis that dynamic network interactions are a key mechanism underlying language network reorganization and recovery.^14,38^

To fill this gap, the present longitudinal study focused on changes in effective connectivity between selected regions within the LN and between regions of the LN and the MDN in patients with aphasia from the acute to the chronic phase after stroke. We employed Dynamic Causal Modelling (DCM) to test for commonalities and differences in effective connectivity across patients with different lesion locations.^39-41^ Our study was based on the functional MRI and behavioural dataset of Stockert *et al*.,^25^ including two groups of patients with either frontal or temporo-parietal stroke in the left hemisphere, as well as a healthy control group. We selected six representative regions of the LN and MDN based on individual functional activation. First, we investigated connectivity patterns in each lesion group at each time point separately. Second, we compared differences between the two lesion groups at each time point. Third, we explored differences between stroke patients and healthy controls. Finally, we correlated language ability and improvement with connectivity parameters to probe the behavioural relevance of different connectivity patterns. We hypothesized that after stroke, across-network interactions between MDN and LN regions as well as within-network interactions between regions of the LN would change over time and depend on lesion location. Based on previous task-related activation studies,^25,27^ increased MDN-to-LN connectivity and right-to-left prefrontal connectivity might already be expected early after stroke while normalization towards left-hemispheric LN interactions might occur later during recovery. We also expected that task-induced modulation of these interactions in the early phase after stroke should correlate with better language ability and improvement.

To the best of our knowledge, our study is the first to examine effective connectivity in patients with post-stroke aphasia longitudinally from the acute to the chronic phase, putting emphasis on the impact of lesion site.

## Materials and methods

### Participants

Two groups of patients with either frontal (*n* = 17, mean age 52.3 ± 18.9 years, 7 female) or temporo-parietal (*n* = 17, mean age 54.4 ± 12.7 years, 2 female) first time ischaemic stroke lesions were examined longitudinally during the acute (≤1 week), subacute (1–2 weeks) and chronic (>6 months) phases after stroke. Patient characteristics are provided in Supplementary Table 1. A control group of 17 healthy age-matched participants (mean age 51.9 ± 19.4, 6 female) was measured once.

### Experimental design

We employed two slightly different versions of a passive auditory sentence comprehension paradigm during functional MRI (fMRI). Both paradigms required participants to listen attentively to short German sentences (auditory speech processing, e.g. ‘The pilot flies the plane’) and temporally reversed versions of the same stimuli (reversed auditory speech processing, e.g. ‘enalp eht seilf tolip ehT’; Supplementary material for details). Language-related activity was obtained by contrasting auditory speech and reversed speech processing.

Language performance was assessed with the Aachen Aphasia Test (AAT),^42^ the standard diagnostic battery for subacute and chronic aphasia in Germany (Supplementary material for details). To assess the relationships between language ability and effective connectivity changes, a language comprehension score based on AAT subtests (auditory and written comprehension and Token Test Scores) was computed separately for each patient and phase. The resulting value between 0 and 1 reflected the level of overall performance, with a score of 1 indicating full recovery (Supplementary Table 2).

### MRI acquisition

Since patients were examined with two slightly different versions of the fMRI paradigm and at different locations, two MRI protocols were used. Functional and structural MRI data from all 51 participants were obtained at a 3 T Siemens TRIO TIM or VERIO MR system (Siemens Medical Systems) with standard imaging protocols (Supplementary material).

Functional MRI preprocessing (slice-timing correction, spatial realignment, coregistration, normalization, resampling and smoothing) was performed with Statistical Parametric Mapping (SPM12; Wellcome Trust Centre for Neuroimaging; http://www.fil.ion.ucl.ac.uk/spm/) implemented in MATLAB (version 8.6). For more information regarding experimental design, stimulus material and fMRI activation analyses, we refer the reader to the Supplementary material from Stockert *et al*.^25^

### Effective connectivity analyses

Effective connectivity within the LN and between the LN and the MDN was explored using DCM with the Parametric Empirical Bayes (PEB) framework in SPM12 implemented in MATLAB (version 9.12). DCM allows investigation of the direct causal influences between a pre-defined set of brain regions. We estimated three sets of parameters: (i) task-independent connectivity from one region of interest (ROI) to another (i.e. the intrinsic connectivity); (ii) direct influences of experimental input on ROI activity (i.e. the driving input); and (iii) task-dependent changes in the connectivity between a pair of ROIs caused by the experimental stimulus (i.e. the modulatory inputs: auditory speech and reversed speech processing).^43^ Our main parameter-of-interest was the modulatory input of auditory speech processing on between-region connectivity.

First, we investigated the effective connectivity for each group in each phase separately. Second, we compared differences between lesion groups in each phase. Finally, the connectivity pattern in each lesion group at each time point was compared with healthy controls. In all analyses, we focused on auditory speech processing-induced modulation of connectivity.

#### Region-of-interest selection

**Figure 1:**
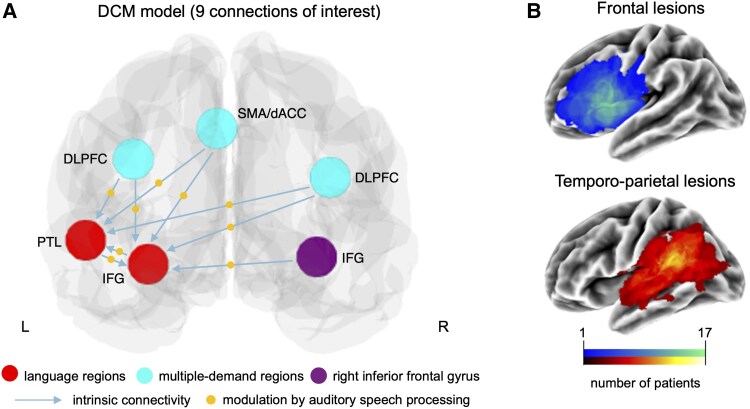
**Illustration of the DCM model parameters of interest and brain lesions.** (**A**) DCM model with parameters of interest only. Auditory speech processing (yellow dots) could modulate each connection of interest (blue arrows). (**B**) Visualization of the left hemisphere lesion distribution in both patient groups (colours represent number of subjects with a lesion in each voxel, adapted from Stockert *et al*.^25^). dACC = dorsal anterior cingulate cortex; DCM = Dynamic Causal Modelling; DLPFC = dorsolateral prefrontal cortex; IFG = inferior frontal gyrus; L = left; PTL = posterior temporal lobe; R = right; SMA = supplementary motor area.

ROIs for the DCM analyses were selected based on the results of our previous fMRI study on the same participants.^25^ In the previous study, 13 regions were defined using activation peaks extracted from the contrast (auditory speech processing > reversed speech processing) across all patients and three time points (cf. Supplementary Table 2 in Stockert *et al*.^25^). In our study, 6 representative ROIs for undamaged areas of the LN (Fig. 1A, red), MDN (Fig. 1A, cyan), and the right-hemispheric homologue of the left inferior frontal gyrus (IFG; Fig. 1A, purple) were selected out of the 13 regions to account for the computational complexity of DCM. The assignment of areas to the LN or MDN was based on *a priori* knowledge about the functional specialization of brain networks.^44-46^ The final set of ROIs representing the LN were in the pars orbitalis in the left IFG [IFGorb, Montreal Neurological Institute (MNI) coordinates *x* = −39, *y* = 29, *z* = −13] and the left posterior temporal lobe [PTL, (−54 −37 2)]. MDN regions included the left and right dorsolateral prefrontal cortex [DLPFC, (−42 2 35), (47 25 28)] and the supplementary motor area/dorsal anterior cingulate cortex [SMA/dACC, (−6 17 50)]. The right IFGorb (42 35 −10) was included as right hemisphere ROI because it showed the strongest activity in the right hemisphere in our previous study.^25^ The right temporal cortex was not considered as a ROI as it did not show significant activation in patients with temporo-parietal lesions.

For each ROI, we created a sphere of 20 mm radius around the mean group coordinate for the contrast (auditory speech processing > reversed speech processing). To prevent time series extraction from lesioned tissue, we first excluded all lesioned voxels from left IFG and DLPFC (for patients with frontal stroke) or left PTL (for patients with temporo-parietal stroke) ROIs. To account for partial lesion volume effects and potential effects of abnormal BOLD signal near the lesion,^47^ perilesional voxels in an area extending from 0 to 3 mm beyond the lesion’s border were additionally excluded. This was achieved by calculating a voxel-wise map of the shortest distance to the lesion.^25^ All voxels were excluded on the individual subject level based on the normalized lesions. To account for individual variability in functional anatomy, in a final step, we defined subject-specific ROIs as the top 10% most activated voxels within each sphere for the contrast (auditory speech processing > reversed speech processing) in each individual subject.^48^

#### First-level DCM model specification

To investigate the effective connectivity in each patient group at each time point and in healthy controls, we performed two-level effective connectivity analyses using DCM and PEB. At the first level, a full model was specified and estimated for each participant, which served as a starting point for Bayesian model reduction. The full model assumed that all ROIs were reciprocally connected. We set the onset of all auditory stimuli as driving input to all ROIs and auditory speech and reversed speech processing as modulatory inputs on the connections between the six ROIs (Supplementary Fig. 1). Following our hypotheses and task-based activation patterns in our previous study,^25^ we focused on the modulatory influence of auditory speech processing on the effective connectivity of nine connections-of-interest (Fig. 1A). These included influences from bilateral MDN regions to left undamaged language areas (SMA/dACC to lIFG, SMA/dACC to lPTL, lDLPFC to lIFG, lDLPFC to lPTL, rDLPFC to lIFG, rDLPFC to lPTL), and from rIFG to lIFG, as well as interactions within the language network (lIFG to lPTL, lPTL to lIFG) (Fig. 1A). The first eigenvariate of the time series was extracted from the subject-specific ROIs and adjusted for ‘effects-of-interest’ (i.e. an F-test across all experimental condition regressors, excluding nuisance regressors). DCM inputs were mean-centred, with the intrinsic connectivity reflecting the mean across all experimental conditions.^40^

#### Second-level Parametric Empirical Bayes analyses

DCM parameters of each subject were entered into group-level PEB models. The PEB model allows the investigation of group effects and between-subjects variability. The initial set of analyses investigated connectivity patterns in each lesion group at each phase separately. Bayesian Model Reduction (BMR) compared hundreds of PEB models with different combinations of parameters switched off (i.e. prior mean and variance set to 0).^39^ Finally, we computed the Bayesian Model Average (BMA), the average of parameter values across models weighted by each model’s probability.

Secondly, we compared differences between lesion groups. We included regressors encoding differences between groups at each time point. Thereafter, the connectivity pattern of each lesion group during each phase was compared with healthy controls. Regressors were added in the PEB model to encode differences between patients and controls.

### Correlations between effective connectivity and language performance

Finally, we investigated the behavioural relevance of the observed interactions. In our previous study, all patients showed language improvement over time.^25^ We reasoned that this improvement might be underpinned by changes in the interactions of the pre-defined brain networks, which should be reflected in correlations between recovery and connectivity.

We thus correlated (changes in) individual language ability with modulatory parameters of auditory speech processing on all nine connections-of-interest with linear mixed models using the lme4 package (version 1.1–31)^49^ in RStudio. Our models aimed to explain subject-specific language ability (‘absolute’ score during each phase) or improvement (score change from one to another phase ‘relative’ to the initial impairment). The improvement in language comprehension scores from the acute to subacute phase was calculated as follows:


(1) Languageimprovement=(subacutescore - acutescore)/(1 - acutescore)

The dividend quantifies the potential for recovery (i.e. the difference from full recovery) to correct for the initial impairment. Language improvement from the acute or subacute to the chronic phase was calculated accordingly (Supplementary Table 2).

To explain language ability during each phase as well as language improvement, we used separate linear mixed models based on the DCM modulatory parameters (Supplementary material for details).

### Statistical analysis

Significant patterns of effective connectivity for the control group and patient groups at each time point were obtained by thresholding the Bayesian Model Average to exclusively retain parameters with a posterior probability larger than 95% (based on Bayesian model comparison with that parameter switched on versus off), which is common practice.^41,48^

The relationships between (changes in) effective connectivity and language performance were examined using linear mixed models. Follow-up linear regressions were conducted if significant interactions were found. Bonferroni correction was applied to adjust for multiple comparisons in follow-up regressions. A corrected threshold of *P* < 0.05 was applied to determine statistical significance for all models.

## Results

### Effective connectivity

We were interested in the influence of auditory speech processing on the connectivity from MDN regions and the right inferior frontal lesion homologue to left-hemispheric language areas, as well as interactions within the LN, and how this pattern depends on post-stroke phase and lesion location. Therefore, we focused on task-induced modulations of the following nine connections-of-interest for all analyses: rIFG to lIFG, SMA/dACC to lIFG, SMA/dACC to lPTL, lDLPFC to lIFG, lDLPFC to lPTL, rDLPFC to lIFG, rDLPFC to lPTL, lIFG to lPTL, and lPTL to lIFG. The full DCM results, including all possible between-region connections, are shown in Supplementary Fig. 2.

#### Control participants

**Figure 2:**
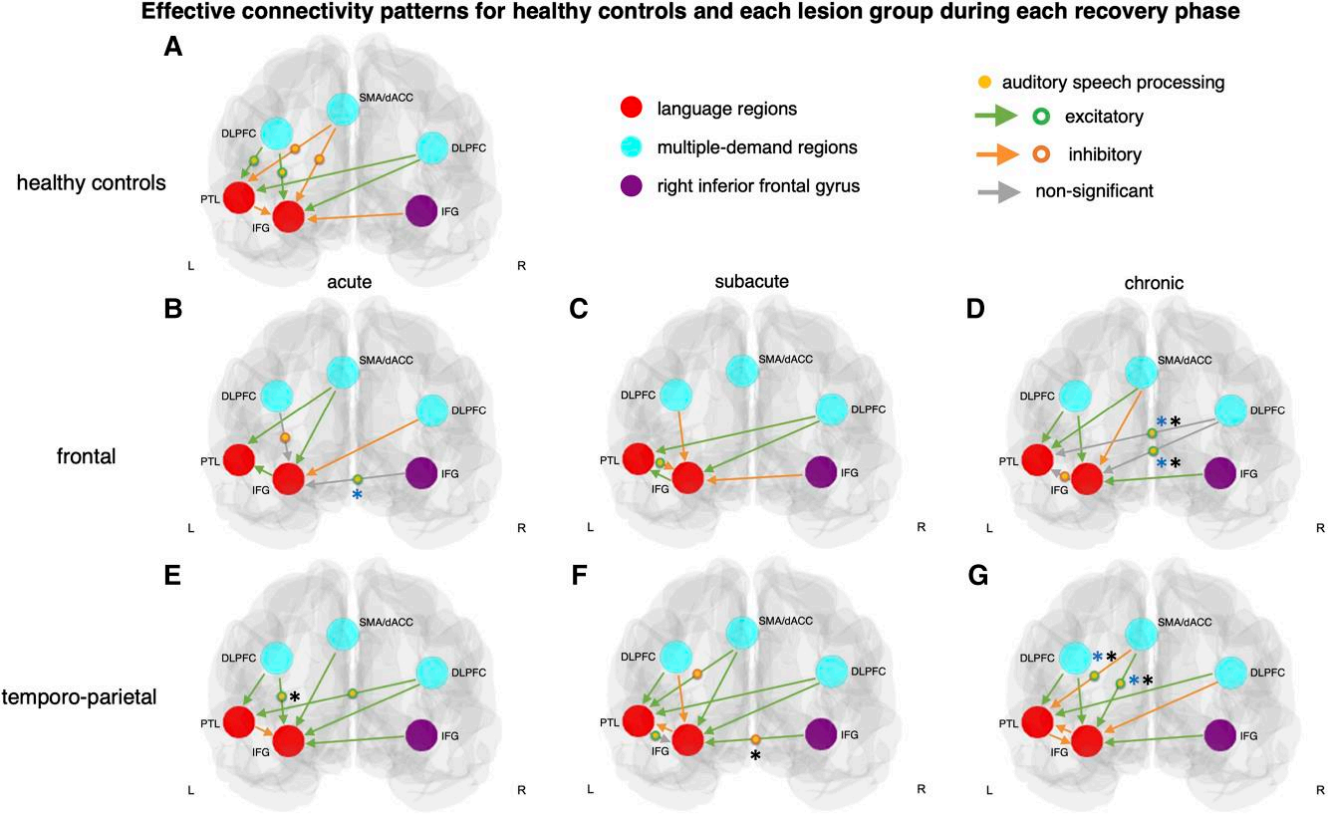
**Effective connectivity patterns for healthy controls and stroke patients.** Effective connectivity patterns for healthy controls (**A**) and patients with left frontal (**B**–**D**) and temporo-parietal (**E**–**G**) lesions in the acute, subacute, and chronic phase. Only significant parameters are displayed (posterior probability larger than 95%). Black asterisks indicate significant differences between lesion groups and blue asterisks between patients and controls (see Supplementary Tables 4 and 5 for further details). Note that intrinsic connectivity can be non-significant (∼zero average connectivity), while modulation by auditory speech processing on the same connection can be significantly positive (facilitatory) or negative (inhibitory). dACC = dorsal anterior cingulate cortex; DLPFC =dorsolateral prefrontal cortex; IFG = inferior frontal gyrus; L = left; PTL = posterior temporal lobe; R = right; SMA = supplementary motor area.

Task-induced modulations of effective connectivity during auditory speech processing in healthy controls are displayed in Fig. 2A and Table 1. The principal pattern was that auditory speech processing significantly facilitated connectivity from the lDLPFC to the lIFG (0.436) and lPTL (0.413). At the same time, auditory speech processing inhibited connectivity from the SMA/dACC to the lIFG (−0.407) and lPTL (−0.384). Significant differences between controls and patients are listed in Supplementary Table 6.

**Table 1:** Parameter estimates of the Bayesian model average for intrinsic connectivity and modulation by auditory speech processing in healthy controls and stroke patients

	Controls		Frontal						Temporo-parietal					
			Acute		Subacute		Chronic		Acute		Subacute		Chronic	
Connections	Intrinsic	Speech	Intrinsic	Speech	Intrinsic	Speech	Intrinsic	Speech	Intrinsic	Speech	Intrinsic	Speech	Intrinsic	Speech
rIFG → lIFG	**−0**.**094**	n.s.	n.s.	**0.501**	**−0**.**123**	n.s.	**0.201**	n.s.	**0**.**156**	n.s.	**0**.**091**	**−0.502**	**0**.**235**	n.s.
SMA/dACC→ lIFG	**−0**.**070**	**−0.407**	**0.092**	n.s.	n.s.	n.s.	**−0.096**	n.s.	**0**.**116**	n.s.	**0**.**086**	n.s.	**0**.**135**	**0.520**
SMA/dACC→ lPTL	**−0**.**159**	**−0.384**	**0.254**	n.s.	n.s.	n.s.	**0.083**	n.s.	n.s.	n.s.	**0**.**110**	**−0.364**	**−0**.**075**	**0.444**
lDLPFC→ lIFG	**0**.**167**	**0.436**	n.s.	**−0.552**	**−0**.**061**	n.s.	**0.141**	n.s.	**0**.**104**	**0.810**	**−0**.**103**	n.s.	**0**.**080**	n.s.
lDLPFC→ lPTL	**0**.**263**	**0.413**	n.s.	n.s.	n.s.	n.s.	**0.295**	n.s.	**0**.**172**	n.s.	**0**.**070**	n.s.	**0**.**143**	n.s.
rDLPFC→ lIFG	**0**.**338**	n.s.	**−0.080**	n.s.	**0**.**264**	n.s.	n.s.	**0.615**	**0**.**195**	n.s.	**0**.**325**	n.s.	**−0**.**071**	n.s.
rDLPFC→ lPTL	**0**.**396**	n.s.	n.s.	n.s.	**0**.**143**	n.s.	n.s.	**0.838**	**0**.**119**	**0.438**	**0**.**081**	n.s.	**0**.**225**	n.s.
lIFG→ lPTL	n.s.	n.s.	**0.122**	n.s.	**0**.**175**	n.s.	n.s.	**−0.726**	n.s.	n.s.	**−0**.**101**	n.s.	**−0**.**147**	n.s.
lPTL→ lIFG	**−0**.**157**	n.s.	n.s.	n.s.	**−0**.**107**	**0.334**	n.s.	n.s.	**−0**.**171**	n.s.	n.s.	**0.354**	**−0**.**083**	n.s.

Positive parameter estimates indicate facilitatory intrinsic connectivity or modulation by auditory speech processing, while negative parameter estimates indicate inhibitory intrinsic connectivity or modulation by auditory speech processing. n.s. = non-significant. All results are thresholded to only include parameters with a posterior probability larger than 95% (bold). Note: the parameter estimates for modulation by auditory speech processing reflect the amount of modulation rather than the resulting connection strength after modulation. dACC = dorsal anterior cingulate cortex; lDLPFC = left dorsolateral prefrontal cortex; lIFG = left inferior frontal gyrus; lPTL = left posterior temporal lobe; rIFG = right inferior frontal gyrus; SMA = supplementary motor area

#### Patients with frontal lesions

In the acute phase of patients with frontal lesions (Fig. 2B), auditory speech processing significantly facilitated the effective connectivity from rIFG to lIFG (0.501). This modulation was relatively stronger compared to healthy controls. In contrast, auditory speech processing inhibited the connection from lDLPFC to lIFG (−0.552). In the subacute phase (Fig. 2C), we observed speech processing-induced facilitation of connectivity from lPTL to lIFG (0.334). In the chronic phase (Fig. 2D), this was followed by speech processing-induced inhibition of connectivity in the opposite direction, from lIFG to lPTL (−0.726). In contrast to controls, auditory speech processing facilitated the influence from rDLPFC to lIFG (0.615) and lPTL (0.838) in the chronic phase. This facilitatory influence of auditory speech processing from the right hemisphere was relatively stronger compared to both patients with temporo-parietal lesions in the chronic phase and controls (Supplementary Tables 5 and 6).

#### Patients with temporo-parietal lesions

In the acute phase of patients with temporo-parietal lesions (Fig. 2E), auditory speech processing significantly facilitated the connectivity from lDLPFC to lIFG (0.810), which was also observed in controls. This modulation was stronger than in patients with frontal lesions in the acute phase (Supplementary Table 3). Additionally, we observed increased facilitatory connectivity from rDLPFC to lPTL (0.438). In the subacute phase (Fig. 2F), comparable to patients with frontal lesions, we found auditory speech processing-induced facilitation from lPTL to lIFG (0.354). In addition, auditory speech processing inhibited connectivity from rIFG to lIFG (−0.502). This inhibitory effect of auditory speech processing was relatively stronger compared to patients with frontal lesions in the subacute phase. In addition, auditory speech processing inhibited connectivity from SMA/dACC to lPTL (−0.364). In the chronic phase (Fig. 2G), contrary to controls and earlier phases, auditory speech processing facilitated connectivity from SMA/dACC to lIFG (0.520) and lPTL (0.444). This facilitatory effect from an alternative MDN region was relatively stronger compared to both patients with frontal lesions in the chronic phase and controls (Supplementary Tables 5 and 6).

### Correlations between effective connectivity and language ability or improvement

Linear mixed model analyses were performed to identify relationships between (changes in) modulatory effects of auditory speech processing on all nine connections-of-interest and individual ability or improvement in language comprehension (Supplementary Table 7).

**Figure 3:**
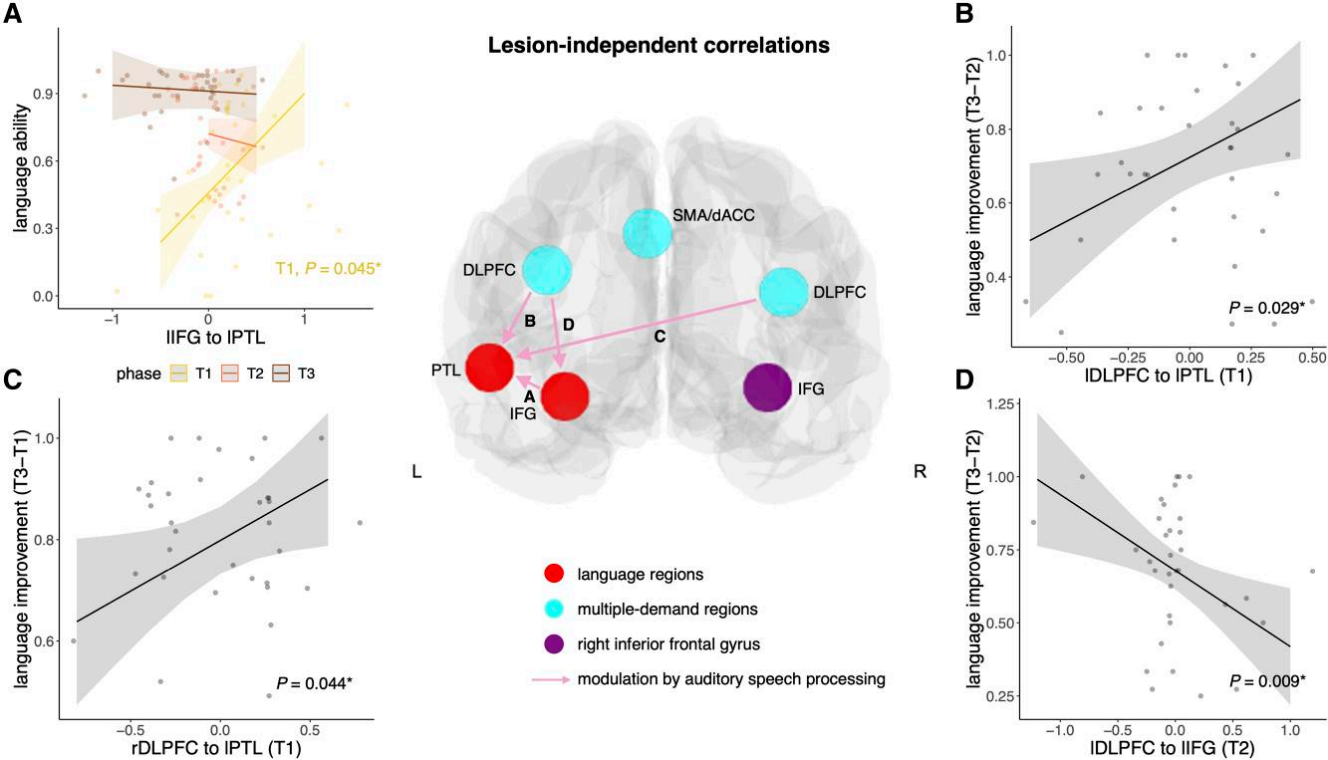
**Lesion-independent relationships between modulation by auditory speech processing and language ability or improvement.** Independent of lesion location, stronger modulation of the connection from lIFG to lPTL at T1 by auditory speech processing was associated with better language ability (**A**). Stronger modulation of the connection from lDLPFC (**B**) and rDLPFC (**C**) to lPTL at T1 by auditory speech processing was associated with more language improvement. Less modulation of the connection from lDLPFC to lIFG at T2 by auditory speech processing correlated with more language improvement (**D**). *Significant effect. T1, yellow = acute phase; T2, orange = subacute phase; T3, brown = chronic phase. Shaded areas represent 95% confidence intervals. dACC = dorsal anterior cingulate cortex; DLPFC = dorsolateral prefrontal cortex; IFG = inferior frontal gyrus; L/l = left; PTL = posterior temporal lobe; R/r = right; SMA = supplementary motor area.

We first report correlations independent of lesion locations, which means that a significant interaction effect with lesion location was absent in the linear mixed models (Fig. 3). For the connection from lIFG to lPTL, we found a two-way interaction between Auditory speech modulation × Phase (*χ^2^* = 11.15, *P* = 0.004). Stronger facilitatory modulation was associated with better language ability during the acute phase (*β* = 0.70, *P* = 0.045) (Fig. 3A). For the connection from lDLPFC to lPTL, greater auditory speech processing-induced facilitation in the acute phase was associated with more language improvement from the subacute to the chronic phase (*β* = 0.35, *P* = 0.029) (Fig. 3B). Similarly, for the contralateral connection from rDLPFC to lPTL, greater facilitatory influence of auditory speech processing in the acute phase was associated with more language improvement from the acute to the chronic phase (*β* = 0.20, *P* = 0.044) (Fig. 3C). In contrast, less task-related modulation of the connection from lDLPFC to lIFG in the subacute phase was associated with more language improvement from the subacute to the chronic phase (*β* = −0.26, *P* = 0.009) (Fig. 3D).

**Figure 4:**
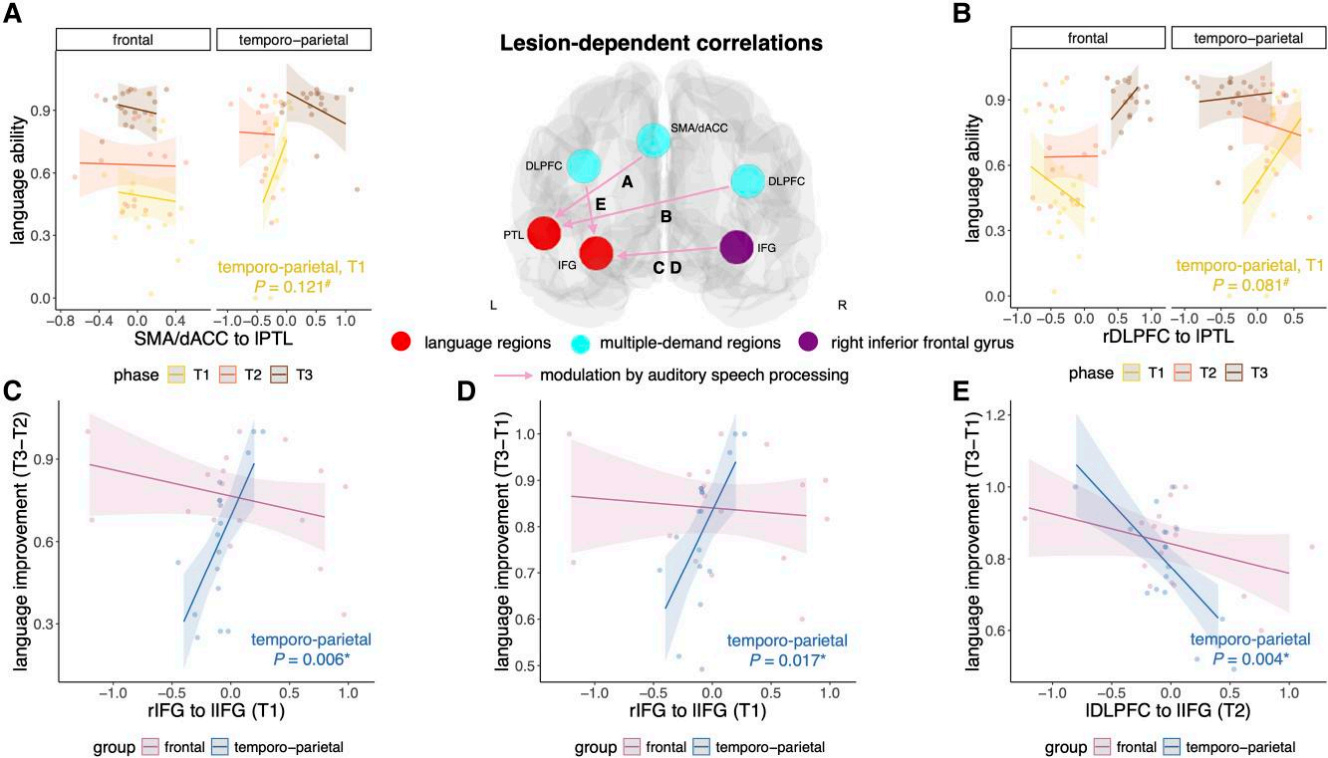
**Lesion-dependent relationships between modulation by auditory speech processing and language ability or improvement.** Stronger modulation of the connection from SMA/dACC (**A**) and rDLPFC (**B**) to lPTL at T1 by auditory speech processing was associated with better language ability. Stronger modulation of the connection from rIFG to lIFG (**C** and **D**) at T1 by auditory speech processing was associated with more language improvement from T1/T2 to T3. Less modulation of the connection from lDLPFC to lIFG at T2 by auditory speech processing was associated with more language improvement (**E**). All relationships were found in patients with temporo-parietal lesions. *Significant effect; ^#^effect does not survive correction for multiple comparisons (i.e. *P* < 0.05 uncorrected). **A** and **B**: T1, yellow = acute phase; T2, orange = subacute phase; T3, brown = chronic phase. **C**–**E**: purple = frontal group; blue = temporo-parietal group. Shaded areas represent 95% confidence intervals. dACC = dorsal anterior cingulate cortex; DLPFC = dorsolateral prefrontal cortex; IFG = inferior frontal gyrus; L/l = left; PTL = posterior temporal lobe; R/r = right; SMA = supplementary motor area.

Secondly, we report associations between modulation by auditory speech processing and language ability or improvement that depend on lesion location. This means that a significant interaction effect of lesion location was found in the linear mixed models (Fig. 4). For the connection from SMA/dACC to lPTL, we found a three-way interaction between Auditory speech modulation × Group × Phase (*χ^2^* = 7.21, *P* = 0.027). Selectively in patients with temporo-parietal stroke, stronger modulation by auditory speech processing was associated with better language ability during the acute phase (does not survive correction for multiple comparisons: *β* = 1.21, *P* = 0.121) (Fig. 4A). This type of interaction was also present for the connection from rDLPFC to lPTL (*χ^2^* = 13.81, *P* = 0.001). Again, selectively in patients with temporo-parietal lesions, stronger modulation by auditory speech processing correlated with better language ability during the acute phase (does not survive correction for multiple comparisons: *β* = 0.83, *P* = 0.081) (Fig. 4B). For the connection from rIFG to lIFG, we found two-way interactions between Auditory speech modulation × Group, where modulation by auditory speech processing in the acute phase was linked to language improvement from the subacute to the chronic phase (*χ^2^* = 17.24, *P* < 0.001) as well as from the acute to the chronic phase (*χ^2^* = 10.79, *P* < 0.001). Specifically, in patients with temporo-parietal lesions, stronger modulation by auditory speech processing in the acute phase was associated with more language improvement from the subacute to the chronic phase (*β* = 0.96, *P* = 0.006) (Fig. 4C) as well as from the acute to the chronic phase (*β* = 0.53, *P* = 0.017) (Fig. 4D). Another two-way interaction between Auditory speech modulation × Group demonstrated that modulation of the connection from lDLPFC to lIFG by auditory speech processing during the subacute phase was linked to language improvement from the acute to the chronic phase (*χ^2^* = 6.89, *P* = 0.009). Specifically, in patients with temporo-parietal lesions, less modulation by auditory speech processing correlated with more language improvement (*β* = −0.36, *P* = 0.004) (Fig. 4E). We found no associations between modulation by auditory speech processing and language ability or improvement specific for frontal lesions.

Third, we report relationships between changes in modulation by auditory speech processing and language improvement. For the connection from rDLPFC to lPTL, independent of lesion group, decreased modulation by auditory speech processing was associated with more language improvement from the acute to the chronic phase (*χ^2^* = 5.450, *P* = 0.020) (Supplementary Fig. 3A). For the connection from lDLPFC to lIFG, we found a two-way interaction between changes in Auditory speech modulation × Group (*χ^2^* = 4.613, *P* = 0.032). Selectively in patients with temporo-parietal stroke, a greater decrease in modulation by auditory speech processing was associated with larger language improvement from the acute to the chronic phase (does not survive correction for multiple comparisons; *β* = −0.19, *P* = 0.092) (Supplementary Fig. 3B). The connectivity patterns of the two patient groups diverged: modulation from rDLPFC to lPTL by auditory speech processing decreased from the acute to the chronic phase in patients with temporo-parietal lesions but increased in patients with frontal lesions. Further analysis revealed an inverse relationship between auditory speech processing-induced facilitation from SMA/dACC to lPTL and facilitation from rDLPFC to lPTL in patients with temporo-parietal lesions (*β* = −0.682, *P* < 0.001) (Supplementary Fig. 4).

In sum, regarding ‘across-network connectivity’, the pattern of healthy controls in terms of facilitatory MDN connectivity from the left DLPFC to regions of the language network (lIFG and lPTL) (Fig. 2A) was altered after focal brain lesions (Fig. 2B–G). Over the time course of recovery, patients employed different MDN regions to modulate language regions: they established a pattern that either mirrored the controls (right DLPFC in patients with frontal lesions; Fig. 2D) or engaged an alternative MDN region (SMA/dACC in patients with temporo-parietal lesions; Fig. 2G). Both the mirrored pattern and engagement of the alternative region contributed to language ability and improvement (Figs 3B and C and 4A and B).

For ‘within-network connectivity’, stronger modulation of the interaction between lIFG and lPTL was beneficial for language ability in the acute phase (Fig. 3A). In the subacute phase, facilitatory connectivity from lPTL to lIFG was present in both lesion groups (Fig. 2C and F), which was switched to inhibitory connectivity in the opposite direction (IFG to lPTL) in the chronic phase only in patients with frontal lesions (Fig. 2D). Additionally, lesion homologue (rIFG) facilitation to lIFG was observed in the acute phase after frontal lesions (Fig. 2B) and correlated with language improvement in patients with temporo-parietal stroke (Fig. 4C and D).

## Discussion

This study is the first longitudinal investigation of task-related network interactions over the course of post-stroke aphasia recovery. We followed patients with either frontal or temporo-parietal lesions from the acute to the chronic phase and focused on across-network interactions between regions of the MDN and LN as well as within-network interactions in the LN. We hypothesized that network interactions change over the course of recovery and depend on lesion location. We also expected that modulation of these interactions by auditory speech processing in the early phase post stroke should correlate with better language ability and improvement.

**Figure 5:**
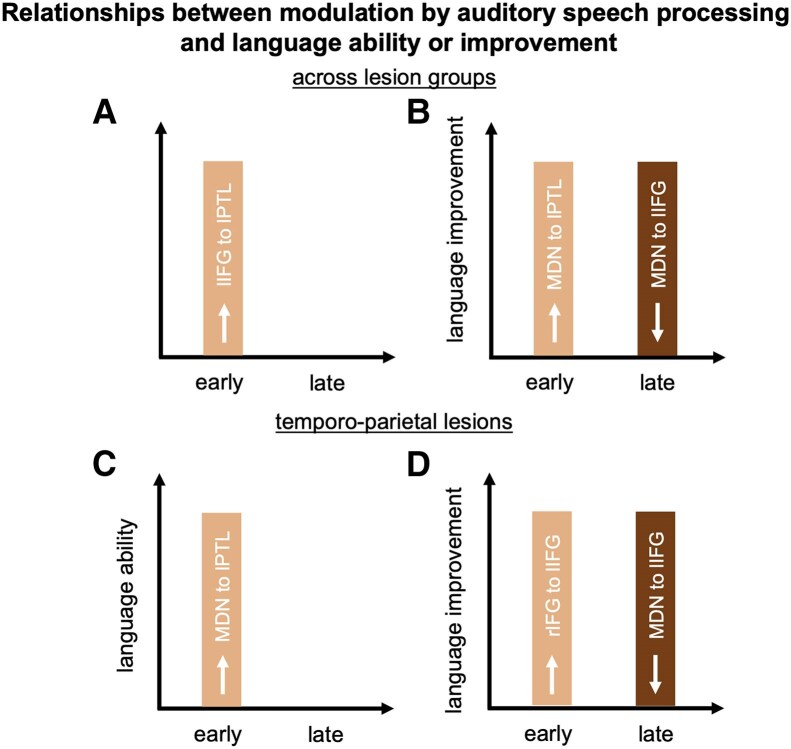
**Schematic relationships between modulation by auditory speech processing and language ability or improvement over the course of recovery**. **A** and **B** display relationships independent of lesion location, and **C** and **D** display relationships selectively for the temporo-parietal group. Early = connectivity in the acute phase, late = connectivity in subacute phase. No relationships were found in the chronic phase and no lesion-specific relationships in the frontal group. IFG = inferior frontal gyrus; l = left; MDN = multiple-demand network; PTL = posterior temporal lobe; r = right.

Our study revealed four main findings. First, successful language recovery requires across-network facilitation from MDN to LN regions. Specifically, patients with frontal lesions showed facilitatory connectivity between regions of the MDN and the LN only in the chronic phase, whereas for patients with temporo-parietal lesions, this was already detectable in the acute phase. Second, the specific MDN region connectivity patterns and their behavioural relevance are not uniform and vary depending on phase post-stroke and lesion location. In the acute phase, facilitatory MDN to LN connectivity was behaviourally beneficial, while throughout recovery, weaker ‘control-like’ MDN to LN connectivity was linked to better long-term language improvement. In the chronic phase, patients with frontal lesions reorganized to a right MDN region that mirrored the pattern of controls, while patients with temporo-parietal lesions engaged the SMA/dACC. Third, within the LN, facilitatory connectivity from lPTL to lIFG transiently emerged in all patients in the subacute phase, which switched to inhibitory connectivity from lIFG to lPTL in the chronic phase in patients with frontal lesions. Fourth, facilitation from the right frontal lesion homologue to lIFG was present during the acute phase in patients with frontal lesions and correlated with language improvement in temporo-parietal stroke. Collectively, our results help disentangle the contribution of different principles of language recovery at the systems level. We provide the first evidence that both (facilitatory) interactions across regions of the MDN and LN and between regions within the LN support language recovery in patients with post-stroke aphasia (Fig. 5). Moreover, our results show that the specific interaction patterns change over the course of recovery and depend on lesion location.

### Across-network connectivity between regions of the multiple-demand and language network

We found facilitation from MDN to LN regions irrespective of lesion location. This converges with earlier reports of increased activation in the bilateral MDN in stroke patients already during less demanding language tasks, congruent with the idea that the MDN supports the LN when the compromised LN itself cannot provide sufficient cognitive resources to master a task.^19,22,50^ This pattern was also present in healthy individuals during auditory speech processing, consistent with prior findings.^6,51^ Whereas all previous studies quantitatively measured activation in the MDN and the LN, our study provides the first evidence for facilitatory influences of MDN on LN regions during language processing in stroke patients and healthy controls. This pattern may indicate that the MDN biases processing in favor of task-relevant information by modulating the response in the LN and allocating domain-general resources such as attention or working memory capacity.^52^ We speculate that in patients in whom parts of the specialized cortices are damaged, reorganization through the formation of new or selection of existing cortical representations in the LN could be supported by the MDN. In this context, we wish to emphasize that the selected regions in the LN were located outside the lesions in all patients, emphasizing the relevance of a strong interaction between both networks.

The observed facilitatory connectivity from MDN to LN regions depends on the phase post-stroke and lesion location. In patients with frontal lesions, this was only detectable in the chronic phase. This may be explained by partial structural disconnection between nodes in the frontal network mediated via the frontal aslant tract connecting left IFG and SMA.^53^ We did not acquire diffusion tensor imaging data in this study; however, future studies should explore the relationship between structural and effective connectivity during language recovery.

In patients with temporo-parietal lesions, the preserved bilateral DLPFC was already able to exert a facilitatory influence over the LN in the acute phase. This complements and extends our fMRI activation studies that revealed an acute global network dysfunction, which resolved in the subacute phase^27^ and was most pronounced in patients with temporo-parietal stroke.^25^ The present results in the same patient sample show that functional interactions already exist in the acute phase despite non-significant group-level activation. Moreover, the significant correlation of early connectivity with later language improvement demonstrates the behavioural relevance of this pattern.

Considering the connectivity pattern of controls, we suggest that in the lesioned brain, the first principle of recovery is an attempt to strengthen pre-existing functional across-network interactions between regions of the LN and the DLPFC. Put differently, during acute stroke, the brain can use existing mechanisms, that is, the allocation of cognitive control to support processing in the preserved LN. Because we did not include a non-linguistic cognitive task, we are unable to distinguish specific cognitive from linguistic processes. Based on the existing literature, the involvement of MDN regions could be interpreted as a response to increased cognitive effort and a compensatory reallocation of cognitive resources due to language impairments. Our observation that, irrespective of lesion location, stronger facilitation from the bilateral DLPFC to lPTL in the acute phase was linked to later language recovery again emphasizes the behavioural relevance of this mechanism. The involvement of the DLPFC in patients could also be explained by greater task difficulty in the presence of more severe impairment during the acute phase.^22^ However, as performance on the passive task with low executive demands did not significantly differ between the acute and subacute or the acute and chronic phase, it is unlikely that this effect can be solely attributed to task difficulty.

Notably, this behavioural benefit of across-network facilitation from MDN to LN regions is not a uniform mechanism over the course of recovery. To the contrary, throughout recovery, decreasing influence of lDLPFC on lIFG and of rDLPFC to lPTL was linked to a favorable outcome and better improvement. This was particularly driven by patients with temporo-parietal stroke and can be interpreted in two ways: partial recovery of the LN may reduce the need for bilateral DLPFC to facilitate processing in undamaged (or partially damaged) language regions; alternatively, as lDLPFC facilitated the lIFG also in controls, this finding could indicate that, as a second principle of recovery, additional regions of the MDN may have substituted the control-like facilitatory mechanism over the course of recovery. This seems plausible when looking at the pattern of MDN regions facilitating LN regions in the chronic phase: in patients with frontal stroke, we found that the right DLPFC facilitated the LN. This can be interpreted as a mirrored pattern relative to controls. Since less language improvement was linked to increased auditory speech processing-induced facilitation from rDLPFC to lPTL, we assume that patients with larger lesions in the left prefrontal cortex turned towards facilitation from rDLPFC in the chronic phase. In contrast, patients with temporo-parietal lesions engaged the SMA/dACC, which inhibited the LN in controls. This finding is further supported by the pattern of an inverse relationship between auditory speech processing-induced modulations on the connection from rDLPFC versus SMA to lPTL in patients with temporo-parietal lesions.

As we did not have a task that differentiates between cognitive functions, we interpret these patterns based on the literature. The bilateral DLPFC is linked to cognitive control processes such as interference suppression and response selection.^12,54,55^ In contrast, SMA/dACC plays a role in monitoring conflicts and detecting salient events.^7,45,56^ Therefore, the observed facilitatory influence of SMA/dACC on language regions in patients with temporo-parietal stroke in the chronic phase may help to resolve competitions among active representations from both auditory speech and reversed speech processing. This may aid the encoding of speech-processing relevant information from auditory input for semantic processing. Patients with temporo-parietal lesions required early cognitive control from the DLPFC for partial recovery in the acute phase and shifted to facilitation from the SMA for selecting semantic representations in the chronic phase. In contrast, patients with frontal lesions, with intact temporal cortices, may not require domain-general support for selecting semantic representations and lacked facilitatory connections from the SMA/dACC to language regions throughout recovery.

Of note, we did not find significant correlations between connectivity and behaviour in the chronic phase. This does not contradict the behavioural relevance of connectivity but could be explained by too little variance of language ability in the chronic phase, as many patients showed full recovery or limited residual deficits despite severe initial impairment (mean language comprehension scores in the chronic phase: 0.91; Supplementary Table 2).

### Within-network connectivity between language regions changes over the course of recovery

Apart from functional interactions between regions of the MDN and LN, post-stroke language reorganization also entails interactions within the language network which again depend on recovery phase and lesion location: in the subacute phase, both lesion groups showed facilitatory auditory speech modulation from lPTL to lIFG, while in the chronic phase, only patients with frontal lesions showed inhibitory modulation from lIFG to lPTL. Previous studies suggested that lPTL is involved in accessing semantic information,^57,58^ while lIFG exerts top-down modulatory control over lPTL to resolve competing semantic representations and suppress task-irrelevant information.^59,60^ As patients attended to meaningful speech during fMRI, we assume that semantic processing took place. In the acute phase, no significant task-related interaction between lIFG and lPTL was observed. This likely reflects the general principle that interactions within the disturbed LN require facilitatory influence from the MDN first. Accordingly, the restoration of facilitatory within-network connectivity between language regions can be regarded as a third principle of recovery. Nevertheless, we found that in the acute phase, a stronger facilitation from lIFG to lPTL was associated with better language ability across groups, which might be driven by patients with less damage in the LN. In line with previous research, this suggests that patients with more fronto-temporal interactions had more preserved language functions.^61^ In the subacute phase, we found evidence for an increase in the bottom-up information flow from lPTL to lIFG which may help to compensate for comprehension impairments after stroke irrespective of lesion location. In the chronic phase, top-down modulatory control from lIFG to lPTL may select task-relevant information and direct the network towards task-specific requirements only in patients with frontal lesions.^62,63^ With respect to within-network connectivity, this could mean that in patients with frontal lesions, lIFG inhibits lPTL to eliminate interference from irrelevant information. In contrast, in patients with temporo-parietal lesions, this supportive mechanism might be missing as representations in the lPTL are impaired and thus cannot be inhibited.

### The beneficial role of prefrontal lesion homologue connectivity

The beneficial versus maladaptive role of the right hemisphere in aphasia recovery is still debated, with most evidence for either direction stemming from studies considering task-related activity only. Several previous studies linked increased task-related activation in the rIFG to better behaviour after left hemisphere stroke, arguing for a supportive role of the prefrontal lesion homologue.^24,27,64,65^ Consistently, our finding of an early facilitatory connectivity increase from the right to left IFG during the acute phase in patients with frontal stroke emphasizes its active contribution to aphasia recovery as a fourth principle of recovery. This further converges with a previous neurostimulation study in healthy volunteers that reported increased facilitatory connectivity from the right to the left IFG during speech repetition immediately after focal perturbation of lIFG.^66^ In that study, increased facilitation was associated with more efficient speech repetition. Although the observed increased facilitatory influence from right to left IFG was stronger in patients with frontal stroke than healthy controls in the present study, we found no behavioural association in these patients. This could reflect the global network dysfunction in the acute phase after stroke, with an overall downregulation of the left IFG that may lead to decreased sensitivity for the facilitatory influence of the right IFG. In contrast, in patients with temporo-parietal lesions, despite an absent group-level effect, increased facilitation from the right to left IFG was associated with better long-term language improvement. This indicates that patients with intact left IFG particularly benefit from stronger inferior frontal interactions in the longer term. Increased task-related connectivity between both prefrontal areas may reflect interactions of executive language processes in the lIFG and cognitive control processes in the rIFG.^28^ This is supported by reports of increased right IFG activation during language comprehension under increasing cognitive demands in healthy volunteers.^67,68^ Similar to MDN regions, rIFG involvement in stroke patients during less demanding language tasks suggests it supports the LN when the LN is compromised. Collectively, these findings indicate an immediate (short-term) contribution of the right IFG to language in patients with frontal stroke and a longer-term role in patients with temporo-parietal stroke.

### Limitations

Several of the observed directed connectivity patterns lacked group-level behavioural relevance. However, the absence of significant correlations does not mean that these reorganization processes are not behaviourally beneficial in stroke patients. Since many patients showed full recovery in the chronic phase, the underlying changes in network interactions, despite being transient, may play a role in facilitating language recovery. Other factors may also have contributed to the lack of behavioural relevance: as we assigned patients to two groups based on their lesion sites, there were ultimately only 17 patients in each group. In addition, lesion heterogeneity may lead to preserved language areas, thus resulting in a different demand for the MDN to support residual language functions.

A general limitation of DCM is its focus on functional interactions within a restricted set of regions rather than large-scale networks. Accordingly, this study examined interactions between representative regions of the LN and MDN, defined based on subject-specific functional activation. Other methods like graph theory^69^ assess functional interactions across large-scale networks. However, these methods evaluate undirected functional interactions (‘functional connectivity’), whereas DCM models directed causal influences (‘effective connectivity’). Future studies should explore large-scale functional connectivity changes during post-stroke aphasia recovery.

Finally, we would like to emphasize that our findings may not generalize to speech production. Future studies should explore the overlap and dissociation in effective connectivity between auditory speech processing and speech production to better understand the impact of processing modality.

## Conclusion

Our longitudinal functional MRI study is the first to elucidate phase- and lesion-dependent effective connectivity patterns in stroke patients over the time course of language recovery. We demonstrate that language recovery relies on increases in task-related interactions both within the language network and across networks. We identified four general principles of language reorganization and uncovered dynamic changes in their behavioural relevance over time. These principles include across-network reorganization, summarizing early and later modulation of the influence from bilateral multiple-demand regions on language regions, as well as within-network reorganization between language regions and early facilitation from the right to the left IFG. Collectively, our findings advance the understanding of the neural mechanisms underlying post-stroke language reorganization, suggesting a dynamic evolution in the relevance of network interactions between language and multiple-demand areas over the time course of recovery.

The identified lesion- and phase-dependent connectivity patterns help inform individualized non-invasive brain stimulation targets for future aphasia rehabilitation. Early after stroke, bilateral MDN regions and the lesion homologue in the right IFG are promising targets for facilitatory stimulation to support across-network reorganization. To strengthen within-network interactions between preserved language areas, facilitatory stimulation of the lPTL in the subacute phase and the lIFG in the chronic phase may enhance reorganization processes. Combining facilitatory stimulation over these areas with speech and language therapy may effectively improve language recovery.

## Supplementary Material

awaf036_Supplementary_Data

## Data Availability

Relevant data, including pseudonymized behavioural and normalized functional MRI data, are publicly available through the figshare repository (doi: 10.6084/m9.figshare.7093481).
