# Supplementary material for: Dynamic reorganization of task-related network interactions in post-stroke aphasia recovery
Source: Brain. 2025 Jan 30;148(10):3563–75. doi: 10.1093/brain/awaf036 (PMC12493039; doi:10.1093/brain/awaf036)
Supplement: awaf036_Supplementary_Data [file awaf036_supplementary_data.pdf]

Supplementary Material

Materials and methods

Experimental design

Apart from auditory speech and reversed speech processing, other types of stimuli (semantically violated speech, e.g., ‘The pilot eats the plane’ in paradigm I; phonotactically legal pseudo speech, e.g., ‘Ren simot plieft mas kugireug’ in paradigm II) were also presented but not used for the contrast of interest. To ensure participants followed the stimuli attentively they were asked to press a button with their left index finger whenever a sentence was incorrect or reversed (paradigm I) or following each stimulus (paradigm II). Task performance did not significantly differ between the acute and subacute phase ($t(31) = -0.89$, $p = 0.381$) or the acute and chronic phase ($t(31) = -1.96$, $p = 0.059$).

Language performance was assessed with the Aachen Aphasia Test (AAT). The AAT is a standardized tool to assess multiple aspects of language function, including spontaneous speech, auditory comprehension, repetition, naming, and writing. The AAT captures both expressive and receptive language abilities and allows to classify different aphasia types as well as aphasia severity.

MRI acquisition

Functional MRI data was acquired using a gradient echoplanar imaging (EPI) sequence (TR = 1.83 or 2.19 s, TE = 25 or 96 ms, flip angle = 70 or 75°, matrix = 64 x 64, field of view 192 mm) with a total of 115 or 260 scans per session with 32 or 36 axial slices covering the whole brain (3 x 3 x 3 mm voxel size, 1 mm slice gap). In the subject-level fMRI activation analysis, we accounted for differences in presentation (six versus one session per examination) by including contrast vector weights which were defined as 1/6 for paradigm I and 1 for paradigm II.

Correlations between effective connectivity and language performance

To explain language ability during each phase, (changes in) DCM modulatory parameters of auditory speech processing, lesion group and recovery phase were added as fixed effects, and random variability across patients was set as random intercept. A three-way interaction (auditory speech modulation * group * phase) was included to test if the relationship between

language ability and auditory speech processing-induced modulation depended on patient groups and/or recovery phases. The linear mixed model was structured as follows:

language ability ~ auditory speech modulation * group * phase + (1 | patients)

To explain language improvement, DCM modulatory parameters of auditory speech processing and lesion group were added as fixed effects, and random variability across groups was set as random intercept. A two-way interaction (auditory speech modulation * group) was also included to test if the relationship depended on patient groups. The linear mixed model was as follows:

language improvement ~ auditory speech modulation * group + (1 | group)

Conditional on significant interactions, stepdown correlation analyses were performed to test which specific group and/or phase(s) drove the interaction.

language ability / language improvement ~ auditory speech modulation

All factors included in the abovementioned models were mean-centered.

Supplementary Figure

Supplementary Figure 1. Illustration of the full DCM model

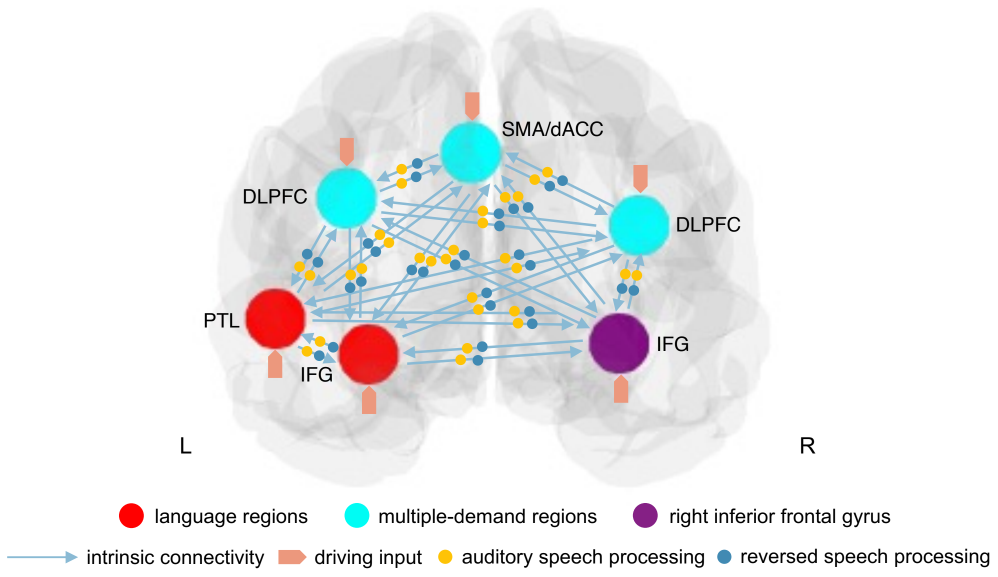

Supplementary Figure 1. Full DCM model, which served as starting point for Bayesian model reduction. All regions were reciprocally connected with each other. Auditory speech processing (yellow dots) and reversed speech processing (blue dots) could modulate each intrinsic connection (light blue arrows), and every region could receive driving input (orange arrow) from all auditory stimuli.

<u>Supplementary Figure 2</u>. Complete effective connectivity patterns for healthy controls and stroke patients

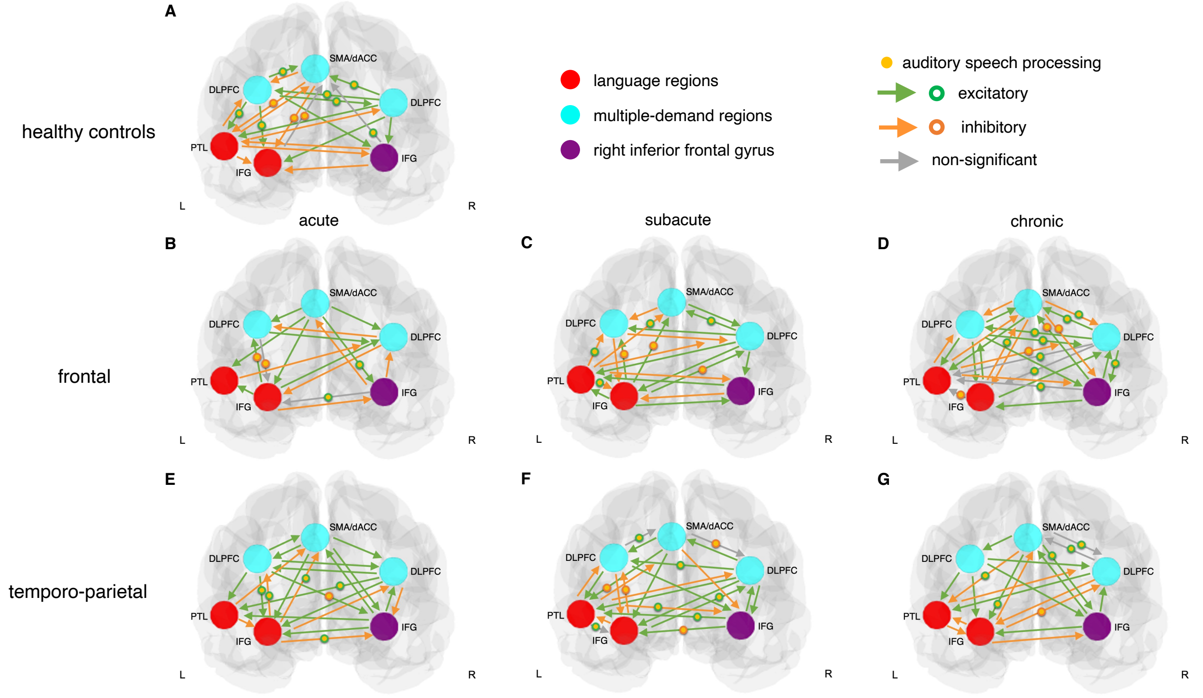

<u>Supplementary Figure 2</u>. Complete effective connectivity patterns for healthy controls **(A)** and patients with left frontal **(B-D)** and temporo-parietal **(E-G)** lesions in the acute, subacute, and chronic phase. Only significant parameters are displayed (posterior probability larger than 95%).

<u>Supplementary Figure 3</u>. Relationships between changes in modulation by auditory speech processing and language improvement

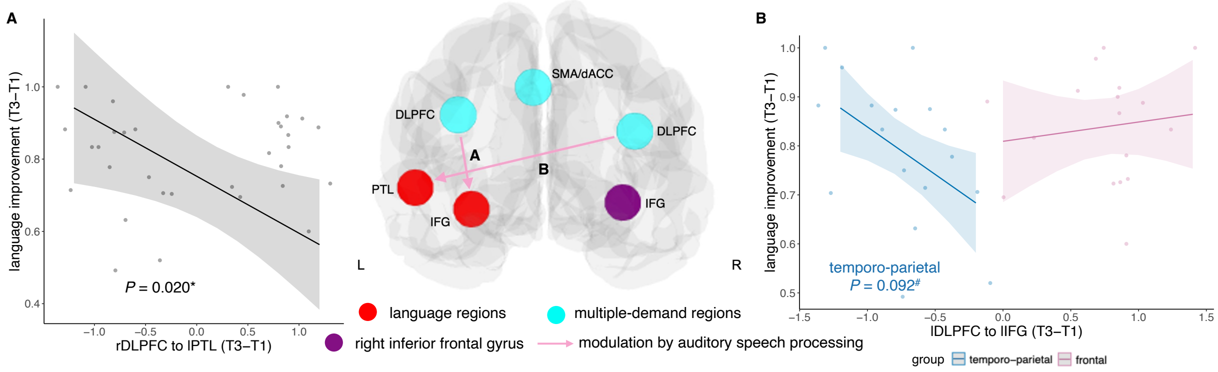

<u>Supplementary Figure 3</u>. **A**. Independent of lesion location decreased modulation of the rDLPFC-to-lPTL connection by auditory speech processing was associated with more language improvement from T1 to T3. **B**. In patients with temporo-parietal lesions, decreased modulation of the lDLPFC-to-lIFG connection by auditory speech processing was associated with more language improvement from T1 to T3. * significant effect; # effect does not survive correction for multiple comparisons (i.e., $p < 0.05$ uncorrected). T1= acute; T2 = subacute; T3 = chronic. **B**. blue = temporo-parietal; purple = frontal. Shaded areas represent 95% confidence intervals.

<u>Supplementary Figure 4</u>. Relationship between modulations of the connection from rDLPFC versus SMA/dACC to lPTL by auditory speech processing in patients with temporo-parietal lesions

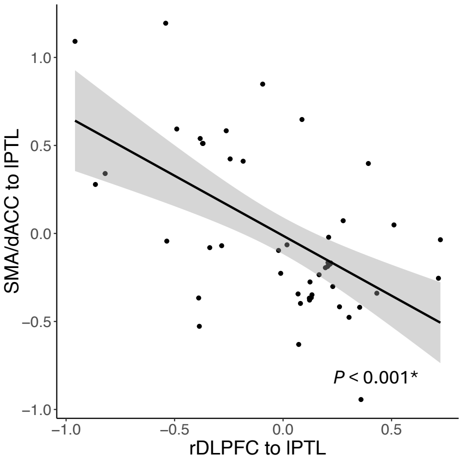

<u>Supplementary Figure 4</u>. In patients with temporo-parietal lesions, modulation of the connection from rDLPFC to lPTL by auditory speech processing is inversely linked to modulation from SMA/dACC to lPTL. * significant effect.

Supplementary Tables

Supplementary Table 1. Patient characteristics

ID	Age	Sex	EHI	fMRI (dpo)			NIHSS	Lesion-volume	Vessel-occlusion	Throm-bolysis
				t1	t2	t3				
**Patients with frontal stroke**										
01	65	M	35	2	15	376	8	12.3	MCA branch	100
02	55	M	10	2	12	396	17	53.2	M1	300
03	16	M	0	1	11	224	16	26.5	MCA branch	130
04	15	M	80	6	17	389	5	71.5	MCA branch	No
05	44	F	100	1	9	219	7	9.0	MCA branch	120
06	26	F	80	6	11	189	20	44.1	distal M1	No
07	52	M	-10	7	12	190	17	22.9	distal M1	100
08	50	F	60	6	13	185	13	53.9	proximal M2	60
09	78	F	-20	3	13	215	2	25.0	MCA branch	No
10	69	M	100	2	15	350	14	60.1	MCA branch	120
11	72	F	100	5	12	281	4	49.4	MCA branch	No
12	40	F	100	2	10	435	13	31.2	ICA dissection[1]	No
13	64	M	80	2	8	275	5	17.2	MCA branch	155
14	62	F	100	4	11	332	20	43.6	M1	180
15	61	M	80	2	10	181	5	42.2	distal M1	No
16	51	M	100	1	12	215	3	41.3	MCA branch	No
17	69	M	100	2	12	182	5	76.6	MCA branch	160

**Mean (STD)**	**52.3 (18.9)**		**64.4 (43.7)**	**3.2 (2.0)**	**11.9 (2.2)**	**272.6 (88.5)**	**10.2 (6.3)**	**69.3 (34.0)**		
**Patients with temporo-parietal stroke**										
18	55	M	100	1	12	352	3	24.4	MCA branch	No
19	37	M	90	2	10	295	7	36.1	MCA branch	175
20	57	M	100	1	10	188	3	6.2	MCA branch	180
21	43	M	100	1	13	204	16	55.5	M1	100
22	39	F	100	4	9	253	21	67.3	M1	No
23	76	M	100	2	12	394	6	108.5	MCA branch	150
24	64	M	100	2	8	270	2	11.9	MCA branch	No
25	61	M	100	1	10	190	1	41.8	MCA branch	No
26	68	m	100	1	10	184	6	32.6	MCA branch	100
27	40	f	100	2	10	190	1	15.8	MCA branch	No
28	49	m	90	2	13	206	4	46.2	ICA dissection[1]	No
29	63	m	100	2	12	364	6	51.5	M1	No
30	69	m	100	2	10	347	2	15.7	ICA[1]	No
31	55	m	60	2	9	368	6	13.4	MCA branch	No
32	58	m	100	1	8	213	1	20.7	ICA[1]	No
33	59	m	100	1	8	213	1	17.4	MCA branch	No
34	31	m	10	1	8	211	2	22.5	MCA branch	No
**Mean (STD)**	**54.4 (12.7)**		**91.2 (23.2)**	**1.6 (0.8)**	**10.1 (1.7)**	**262.5 (75.0)**	**5.2 (5.5)**	**54.8 (41.1)**		

Supplementary Table 1. EHI, hand dominance according to Edinburgh Handedness Inventory (Oldfield, 1971); fMRI (dpo), time of functional MRI examination in days post-stroke onset; NIHSS, National Institutes of Health Stroke Scale at symptom onset; lesion volume in ml, thrombolysis in minutes post symptom onset; vessel occulusion: ICA, internal carotid artery, MCA, middle cerebral artery; M1, sphenoidal segment of the middle cerebral artery. [1]ICA recanalization at follow-up duplex ultrasound examination.

Supplementary Table 2. Language comprehension score and language improvement score for each patient

ID	Language comprehension score			Language improvement score		
	**T1**	**T2**	**T3**	**T2-T1**	**T3-T2**	**T3-T1**
01	0.51	0.58	0.96	0.14	0.90	0.92
02	0.29	0.41	0.81	0.17	0.68	0.73
03	0.27	0.44	0.92	0.23	0.86	0.89
04	0.98	1.00	1.00	1.00	1.00	1.00
05	0.43	0.68	0.95	0.44	0.84	0.91
06	0.34	0.66	0.89	0.48	0.68	0.83
07	0.59	0.69	0.91	0.24	0.71	0.78
08	0.02	0.42	0.89	0.41	0.81	0.89
09	0.40	0.45	0.89	0.08	0.80	0.82
10	0.18	0.40	0.75	0.27	0.58	0.70
11	0.42	0.87	1.00	0.78	1.00	1.00
12	0.35	0.44	0.82	0.14	0.68	0.72
13	0.80	0.97	0.98	0.85	0.33	0.90
14	0.55	0.65	0.99	0.22	0.97	0.98
15	0.85	0.86	0.98	0.07	0.86	0.87
16	0.38	0.47	0.83	0.15	0.68	0.73
17	0.85	0.88	0.94	0.20	0.50	0.60
18	0.94	0.97	1.00	0.50	1.00	1.00
19	0.84	0.94	0.98	0.63	0.67	0.88
20	0.83	0.92	0.98	0.53	0.75	0.88
21	0.94	1.00	1.00	1.00	1.00	1.00
22	0.73	0.89	0.92	0.59	0.27	0.70
23	0.83	0.92	0.98	0.53	0.75	0.88
24	0.52	0.68	0.88	0.33	0.63	0.75
25	0.76	0.92	0.96	0.67	0.50	0.83
26	0.86	0.93	0.96	0.50	0.43	0.71
27	0.00	0.48	0.96	0.48	0.92	0.96
28	0.00	0.36	0.52	0.36	0.25	0.52
29	0.37	0.56	0.68	0.30	0.27	0.49
30	0.91	0.97	0.98	0.67	0.33	0.78
31	0.13	0.59	0.89	0.53	0.73	0.87
32	0.66	0.79	0.90	0.38	0.52	0.71
33	0.81	0.84	0.93	0.16	0.56	0.63
34	0.58	0.62	0.93	0.10	0.82	0.83
**Mean**	**0.56**	**0.71**	**0.91**	**0.42**	**0.68**	**0.81**

Supplementary Table 2. Language comprehension score was computed based on subtests of Aachen Aphasia Test (AAT, auditory and written comprehension and Token Test Scores). The resulting range between 0 and 1 reflected the level of overall performance, with a score of 1 representing full recovery. The improvement in language comprehension scores from the acute

to subacute phase was calculated via the following formula: language improvement = (subacute score - acute score) / (1 – acute score). The dividend quantifies the potential for recovery (i.e., the difference from full recovery) to correct for the initial impairment. Language improvement from the subacute to the chronic phase as well as from the acute to the chronic phase were calculated in the same way. T1 = acute, T2 = subacute, T3 = chronic.

Supplementary Table 3. Anatomical location of regions-of-interest (ROIs)

ROI	Macroanatomy assigned based on Maximum Probability Map
left DLPFC	24.4% in precentral gyrus;<br>15.2% in middle frontal gyrus
left IFG	21.8% in frontal orbital cortex;<br>12.6% in frontal pole
left PTL	13.7% in middle temporal gyrus;<br>10.2% in planum temporale
SMA/dACC	26.5% in superior frontal gyrus;<br>15.3% in paracingulate gyrus
right DLPFC	23.1% in middle frontal gyrus;<br>8.2% in inferior frontal gyrus, pars opercularis
right IFG	24.7% in frontal pole;<br>14.8% in frontal orbital cortex

Supplementary Table 3. Overlaps between 20mm sphere for each ROI and anatomical cluster were calculated using SPM Anatomy toolbox (version 3.0; https://www.fz-juelich.de/en/inm/inm-7/resources/jubrain-anatomy-toolbox). Note that not all cortical regions are currently provided in the toolbox.

Supplementary Table 4. Overlaps between lesions and regions-of-interest (ROIs)

ID	ROI	Number of lesioned voxels	Number of undamaged voxels	Percentage of lesioned voxels (%)	Number of voxels after removal of lesions and perilesional areas	Number of top 10% most activated voxels
**Patients with frontal lesions**						
01	left DLPFC	157	1080	12.69	1065	107
	left IFG	13	1224	1.05	770	77
02	left DLPFC	162	1075	13.10	924	92
	left IFG	39	1198	3.15	740	74
03	left DLPFC	589	648	47.62	478	48
	left IFG	1	1236	0.08	632	63
04	left DLPFC	668	569	54.00	358	36
	left IFG	162	1075	13.10	599	60
05	left DLPFC	153	1084	12.37	1069	107
	left IFG	27	1210	2.18	738	74
06	left DLPFC	199	1038	16.09	824	82
	left IFG	0	1237	0.00	764	76
07	left DLPFC	45	1192	3.64	1162	116
	left IFG	649	588	52.47	276	28
08	left DLPFC	136	1101	10.99	1086	109
	left IFG	420	817	33.95	458	46
09	left DLPFC	521	716	42.12	701	70
	left IFG	649	588	52.47	268	27
10	left DLPFC	92	1145	7.44	1057	106
	left IFG	409	828	33.06	402	40
11	left DLPFC	505	732	40.82	717	72
	left IFG	400	837	32.34	486	49
12	left DLPFC	940	297	75.99	113	11
	left IFG	574	663	46.40	240	24
13	left DLPFC	1109	128	89.65	15	2
	left IFG	518	719	41.88	250	25
14	left DLPFC	6	1231	0.49	1057	106
	left IFG	301	936	24.33	416	42
15	left DLPFC	418	819	33.79	660	66
	left IFG	681	556	55.05	200	20
16	left DLPFC	0	1237	0.00	1032	103
	left IFG	129	1108	10.43	626	63
17	left DLPFC	231	1006	18.67	830	83
	left IFG	136	1101	10.99	582	58
**Patients with temporo-parietal lesions**						
18	left PTL	417	820	33.71	779	78
19	left PTL	37	1200	2.99	1117	112
20	left PTL	23	1214	1.86	1052	105
21	left PTL	83	1154	6.71	1071	107
22	left PTL	74	1163	5.98	1029	103
23	left PTL	369	868	29.83	624	62
24	left PTL	465	772	37.59	721	72
25	left PTL	282	955	22.80	652	65

26	left PTL	488	749	39.45	585	59
27	left PTL	447	790	36.14	611	61
28	left PTL	108	1129	8.73	1046	105
29	left PTL	511	726	41.31	376	38
30	left PTL	104	1133	8.41	987	99
31	left PTL	104	1133	8.41	1064	106
32	left PTL	422	815	34.11	499	50
33	left PTL	609	628	49.23	378	38
34	left PTL	491	746	39.69	491	49

Supplementary Table 4. We calculated the number and percentage of lesioned voxels within the 20mm spheres for the left DLPFC and IFG ROI in each patient with frontal lesions, as well as for the left PTL in each patient with temporo-parietal lesions. We excluded all lesioned voxels and perilesional voxels in an area extending from 0 to 3 mm beyond the lesion's border. To account for individual variability in functional anatomy, we defined subject-specific ROIs as the top 10 % most activated voxels within each sphere for the contrast [auditory speech processing > reversed speech processing] in each individual subject.

Supplementary Table 5. Parameter estimates encoding differences in modulation by auditory speech processing between lesion groups at each post-stroke phase

	Temporo-parietal vs Frontal		
**Connections**	**Acute**	**Subacute**	**Chronic**
**rIFG to lIFG**	n.s.	**-0.211**	n.s.
**SMA/dACC to lIFG**	n.s.	n.s.	**0.358**
**SMA/dACC to lPTL**	n.s.	n.s.	**0.332**
**lDLPFC to lIFG**	**0.579**	n.s.	n.s.
**lDLPFC to lPTL**	n.s.	n.s.	n.s.
**rDLPFC to lIFG**	n.s.	n.s.	**-0.375**
**rDLPFC to lPTL**	n.s.	n.s.	**-0.558**
**lIFG to lPTL**	n.s.	n.s.	n.s.
**lPTL to lIFG**	n.s.	n.s.	n.s.

Supplementary Table 5. Positive parameter estimates indicate stronger modulation by auditory speech processing in stroke patients with temporo-parietal lesions than frontal lesions, while negative parameter estimates indicate stronger modulation by auditory speech processing in patients with frontal lesions than temporo-parietal lesions. n.s. refers to non-significant difference. All results are thresholded to only include parameters with a posterior probability larger than 95% (bold).

Supplementary Table 6. Parameter estimates encoding differences in modulation by auditory speech processing between healthy controls and stroke patients in each group at each phase

	Frontal vs Control			Temporo-parietal vs Control		
**Connections**	**Acute**	**Subacute**	**Chronic**	**Acute**	**Subacute**	**Chronic**
**rIFG to lIFG**	**0.286**	n.s.	n.s.	n.s.	n.s.	n.s.
**SMA/dACC to lIFG**	n.s.	n.s.	n.s.	**0.393**	**0.264**	**0.375**
**SMA/dACC to lPTL**	**0.238**	n.s.	**0.271**	n.s.	n.s.	**0.350**
**lDLPFC to lIFG**	**-0.437**	n.s.	n.s.	n.s.	n.s.	n.s.
**lDLPFC to lPTL**	**-0.360**	n.s.	**-0.317**	**-0.271**	n.s.	n.s.
**rDLPFC to lIFG**	n.s.	n.s.	**0.284**	n.s.	n.s.	n.s.
**rDLPFC to lPTL**	n.s.	n.s.	**0.401**	n.s.	n.s.	n.s.
**lIFG to lPTL**	n.s.	n.s.	**-0.378**	n.s.	n.s.	n.s.
**lPTL to lIFG**	n.s.	n.s.	n.s.	n.s.	n.s.	n.s.

Supplementary Table 6. Positive parameter estimates indicate stronger modulation by auditory speech processing in stroke patients than healthy controls, while negative parameter estimates indicate stronger modulation by auditory speech processing in healthy controls than stroke patients. n.s. refers to non-significant difference. All results are thresholded to only include parameters with a posterior probability larger than 95% (bold).

Supplementary Table 7. Association of language ability and modulation by auditory speech processing

Language comprehension score		
**rIFG to lIFG**	modulation by auditory speech processing	χ2 = 0.138, p = 0.710
	auditory speech modulation * group	χ2 = 0.521, p = 0.470
	auditory speech modulation * phase	χ2 = 0.332, p = 0.847
	auditory speech modulation * group * phase	χ2 = 0.716, p = 0.699
**SMA/dACC to lIFG**	auditory speech modulation	χ2 = 0.704, p = 0.401
	auditory speech modulation * group	χ2 = 1.868, p = 0.172
	auditory speech modulation * phase	χ2 = 2.248, p = 0.325
	auditory speech modulation * group * phase	χ2 = 3.583, p = 0.167
**SMA/dACC to lPTL**	auditory speech modulation	χ2 = 0.941, p = 0.332
	auditory speech modulation * group	χ2 = 3.820, p = 0.051
	auditory speech modulation * phase	**χ2 = 6.983, p = 0.030**
	auditory speech modulation * group * phase	**χ2 = 7.213, p = 0.027**
**lDLPFC to lIFG**	auditory speech modulation	χ2 = 0.072, p = 0.789
	auditory speech modulation * group	χ2 = 0.173, p = 0.678
	auditory speech modulation * phase	χ2 = 0.127, p = 0.938
	auditory speech modulation * group * phase	χ2 = 0.122, p = 0.941
**lDLPFC to lPTL**	auditory speech modulation	χ2 = 0.088, p = 0.767
	auditory speech modulation * group	χ2 = 1.622, p = 0.203
	auditory speech modulation * phase	χ2 = 0.731, p = 0.694
	auditory speech modulation * group * phase	χ2 = 3.536, p = 0.171
**rDLPFC to lIFG**	auditory speech modulation	χ2 = 0.721, p = 0.396
	auditory speech modulation * group	χ2 = 0.985, p = 0.321
	auditory speech modulation * phase	χ2 = 0.190, p = 0.910
	auditory speech modulation * group * phase	χ2 = 0.037, p = 0.982
**rDLPFC to lPTL**	auditory speech modulation	χ2 = 2.533, p = 0.112
	auditory speech modulation * group	χ2 = 0.639, p = 0.424
	auditory speech modulation * phase	χ2 = 3.712, p = 0.156
	auditory speech modulation * group * phase	**χ2 = 13.81, p = 0.001**
**lIFG to lPTL**	auditory speech modulation	χ2 = 1.968, p = 0.161
	auditory speech modulation * group	χ2 = 3.228, p = 0.072
	auditory speech modulation * phase	**χ2 = 11.15, p = 0.004**
	auditory speech modulation * group * phase	χ2 = 4.622, p = 0.099
**lPTL to lIFG**	auditory speech modulation	χ2 = 0.335, p = 0.562
	auditory speech modulation * group	χ2 = 0.013, p = 0.908
	auditory speech modulation * phase	χ2 = 0.418, p = 0.811
	auditory speech modulation * group * phase	χ2 = 0.303, p = 0.859

Supplementary Table 7. Relationships between language ability and modulation by auditory speech were examined using linear mixed models. To explain the *absolute* language comprehension score, DCM modulatory parameters of auditory speech processing, lesion group and recovery phase were added as fixed effects, and random variability across patients was set as

random intercept. A three-way interaction (auditory speech modulation * group * phase) was included to test if the relationship between language ability and modulation by auditory speech processing depended on patient groups and/or recovery phases. The linear mixed model was structured as follows: language comprehension score ~ auditory speech modulation * group * phase + (1 | patients). Statistical significance was defined by $p < 0.05$ (bold).
